# Behind and beyond the pediatric metabolic syndrome

**DOI:** 10.1186/1824-7288-35-41

**Published:** 2009-12-22

**Authors:** Paolo Brambilla, Angelo Pietrobelli

**Affiliations:** 1 ASL Milano 2, Milano, Italy; 2 FIMP Federazione Italiana Medici Pediatri, Dipartimento Formazione Permanente, Lombardia, Italy; 3 Pediatric Unit, Verona University Medical School, Verona, Italy

## Abstract

The growing use of the Metabolic Syndrome in pediatric age need a critical approach, on the basis of recent concerns on definition and usefulness for individual management in clinical practice. We reviewed these aspects from a pediatric point of view, providing a set of questions about what the Metabolic Syndrome means in a clinical setting. The new proposed pediatric definition by IDF was discussed, by outlying how it does not fully consider the peculiarities of children and adolescents. The comparison between two cases of obese children was used in order to show how this diagnosis could be confusing for a correct management. We stressed the need for health-related limits for each component of the Metabolic Syndrome instead of percentile-derived cut-points, as well as the opportunity to extend the estimation to other family or individual risk factors by means of a multiple-items screening form. In conclusion, Metabolic Syndrome use in pediatric age suffers at present from important limitations (i.e., adult derived definition, possibility to rule-in but not to rule-out the individual metabolic risk, instability of MetS during adolescence, poor usefulness of the diagnosis for specific treatment). Consequently, a prudent use of Metabolic Syndrome for children and adolescents seems to be the best and honest position for paediatricians, waiting for long term, longitudinal follow-up studies that could clarify the entire question.

## Introduction

A recent scientific statement from the American Heart Association and other related Committees focussed the topic of the Metabolic Syndrome (MetS) in pediatric age [1]. The paper provided a set of fundamental questions about what the pediatric MetS means in clinical or research setting. The Authors concluded defining limits of our current knowledge and providing suggestions for needed future research [1]. This position paper outlined most of the concerns that pediatricians feel about the usefulness of MetS diagnosis in day by day clinical practice. Concerns also are referred to which MetS definition has to be used for children. Controversies are mainly related to two diverging approaches: one adapting the definition of MetS from adults [2-4], the other considering the peculiarities of children and adolescents [5,6].

Aim of the present paper is to discuss difficulties found by pediatricians facing to MetS definition and usefulness.

## Discussion

### Metabolic syndrome definition

Metabolic syndrome is the clustering of specific metabolic abnormalities found in overweight and obese subjects, but present also in some normal weight subjects. In adults, the presence of three among clinical (obesity or abdominal obesity, hypertension) and metabolic parameters (hyperglycemia, high triglycerides, low HDL-cholesterol) is used to define MetS [2-4]. In the last decade, many studies in paediatrics derived MetS definition from those used for adults, mostly adapting cut-off points for each parameter to children or adolescents. For this purpose, the percentile methodology was generally used. Recently, the International Diabetes Federation (IDF) proposed a new pediatric definition according to age groups [7]. In particular, IDF suggested that the MetS should not be diagnosed in children younger than 10 yrs, while for subjects 10 to 16 years they proposed the use of adult IDF MetS definition [3] with the only difference represented by waist 90^th ^percentile instead of absolute values. Therefore, the IDF defined a 10-16 yrs old subject as having the MetS if waist circumference was ≥ the 90^th ^percentile and if two other of the following items were above or under a single cut-point: triglycerides ≥ 150 mg/dl, HDL < 40 mg/dl, glucose ≥ 100 mg/dl, systolic or diastolic blood pressure (BP) ≥ 130 or ≥ 85 mmHg, respectively [7].

Despite the pediatric IDF definition is becoming to be used in epidemiological studies [8,9], pediatricians face these proposed new limits. Glucose cut-point corresponds to that proposed by the American Diabetes Association few years ago with a general agreement [10], as well as the 90^th ^waist circumference percentile is a worldwide considered and accepted limit [11]. On the contrary, the use of single cut-points for high triglycerides and low HDL, independently from the known age- and gender-related variations in this age group, is particularly striking due to the pubertal impact on lipid parameters [12].

### Hypertension diagnosis in pediatric age

The most discussed limit among those proposed by IDF is the single blood pressure (BP) cut-point (i.e., 130 for systolic or 85 for diastolic) instead of those related to age, gender and height. Up to now, the risk for hypertension was generally set at the 95^th ^percentile level according to National Health Blood Pressure Education Program (NHBPEP) chart [13]. It appears that the difference between the two limits can be conspicuous, especially for younger subjects with lowest height percentiles, and therefore that a certain degree of underestimation of hypertension could derive from the application of IDF proposal. The main question is whether the pediatrician should prefer the use of single BP limit (130/85), derived from adult clinical surveys and linked to health consequences [14], instead of the use of multiple (up to 84 couple of values in this age interval) age, gender, and height-related limits [13]. However, the latter cumbersome approach seems to be justified by the known variation of BP values in pediatric subjects due to the mentioned variables. At the same time it is striking that an unique BP limit for hypertension risk could work in adult population with subjects of different ages and height ranging even from 150 to 200 cm. In other words, is there an excessive complication of hypertension diagnosis by pediatricians, or a simplification of it for adult doctors, or maybe both? The question remains open. Recently, the discussion on hypertension diagnosis in pediatrics was stimulated by the publication of a simplified table of BP values to be used for screening hypertension risk. This proposal suggested to use, for each age group and gender, the 90^th ^percentile usually applied for shorter children (5^th ^height percentile) [15] in order to avoid undiagnosed hypertension, an approach which seems to take the opposite direction than that of the IDF definition [7]. At the same time, a recent paper together with the accompanying editorial [16,17] rose other doubts on pediatric hypertension diagnosis, up to now based on normative values instead of on health-related ones. In any case, until this point will be clarified by further longitudinal surveys, it seem preferable for pediatric hypertension diagnosis the use of the more prudent NHBPEP strategy instead of the IDF proposal [7].

### Metabolic Syndrome usefulness in clinical practice

In Table 1 we show comparison between 2 obese boys 12 years old with similar excessive BMI and increased waist circumference. The diagnosis of MetS can be done only in the case #1 according to the pediatric definition [7], while in the case #2 we can found the presence of only 2 abnormal parameters. Nevertheless, if we look to other variables not included in the set for MetS, we note that the case #2 shows a clustering of both familial and individual factors which can be considered closely associated with metabolic risk. Recently it was demonstrated the relevant importance of positive family history for type 2 diabetes and other CVD-related diseases in the prediction of future health and risk for MetS [18,19]. Therefore, we feel that we cannot estimate an higher risk for case #1 respect to case #2 on the basis of the presence of MetS diagnosis alone. Even in the absence of an overt metabolic pattern, as represented by the MetS, the paediatrician should extend the analysis by looking for the presence of other factors (familial or individual) which could confer to that child a potential future risk. We should also highlight the limitations of a dichotomous definition of the MetS, by preferring a continuous indicator [6,20], or the use of a multiple-items screening form [5].

**Table 1:** Comparison between 2 cases of 12 years old boys for parameters related to metabolic syndrome and other family or individual risk factors.

	Case #1	Case #2
BMI*	26.5	26.5

Waist circumference** (cm)	**86**	**85**

Glucose (mg/dl)	86	85

Triglycerides (mg/dl)	142	115

HDL cholesterol (mg/dl)	**38**	42

Blood pressure (mmHg)	**136/**78	132/**86**

MetS diagnosis, according to [7]	yes	No

Family history for CVD related diseases (in 1^st ^or 2^nd ^degree)	negative	DM2, hypertension, dyslipidemia

Birth weight status	normal	small for gestational age

Early adiposity rebound	no	yes (at 4 years of age)

*BMI as obese according to Cole et al [30]

** Waist circumference values > 90^th ^percentile according to Fernandez et al [11]

Values over or under the cut-points for MetS according to Zimmet et al [7] are reported in bold character.

Taking into consideration MetS diagnosis, we could suggest that its presence in a child could be useful for rule-in but not for rule-out a risk for future health. In fact, there is the possibility of having a false negative estimation in those children not (yet) achieving MetS diagnosis but having other potential factors. In other words, the presence of one cardiovascular risk factor should raise suspicion that additional risk factors may also be present and encourage further investigation [21]. Moreover, it has been proposed that hepatic steatosis should be considered as an additional diagnostic criterion to be added in future approach to this problem [22].

On the other hand we need to consider the clinical value of MetS diagnosis for subject's management. MetS can be considered the clustering of specific conditions and metabolic disorders, but none of them is required to be clinically severe. In fact, the diagnosis can be done by the presence of an increased abdominal adiposity together with pre-hypertension or mild lipid abnormalities, and does not necessarily imply the presence of hypertension, dyslipidemia or reduced glucose tolerance, diseases for which a specific diagnosis is already available. As a consequence of this statement, no specific treatment is strictly necessary in order to manage these findings, except for lifestyle and nutritional changes needed for a correction of excess weight. Therefore, the MetS diagnosis seems not useful for individual treatment, but it can be considered helpful for understanding the possible underlying pathogenesis and for addressing further investigations. Emphasis should be given to the presence of physical inactivity when aiming to estimate the individual metabolic risk, as an impressive mass of data are now confirming the effect of the degree of physical fitness for its prevention in childhood [23-27].

Finally, a certain degree of instability has been described in adolescents with MetS during a 3-year observational study in which almost half of previously affected subjects lost their condition [28]. Another recent study confirmed the MetS variability in pediatric age [29].

## Conclusions

We must support the need for consensus and further studies on MetS, as stated by the American Heart Association [1]. The main questions to be solved are: 1) is it really safe that a child younger than 10 years should not be diagnosed? 2) which is the clinical cost of considering a 10-16 years adolescent like an adult? 3) is it mandatory to identify cut-points related with future health risk instead of normative ones in pediatric age?

Until these points will be ascertained, we strongly suggest to face the topic of MetS taking into consideration that, at present time, MetS in pediatric age suffers from important limitations (i.e., adult derived definition, possibility to rule-in but not to rule-out the individual metabolic risk, instability of MetS during adolescence, poor usefulness of the diagnosis for specific treatment).

We think that this should be the best and honest position that a paediatrician can now apply to MetS in childhood, waiting for long term, longitudinal follow-up studies that could clarify the entire question.

## List of Abbreviations used

MetS: metabolic syndrome; HDL: high density lipoprotein cholesterol; IDF: International Diabetes Federation; BP: blood pressure; NHBPEP: National Health Blood Pressure Education Program; CVD: cardiovascular disease; BMI: body mass index; DM2: type 2 diabetes;

## Competing interests

The authors declare that they have no competing interests.
